# The size criteria in minimally invasive video-assisted thyroidectomy

**DOI:** 10.1186/1471-2482-7-2

**Published:** 2007-01-25

**Authors:** Massimo Ruggieri, Andrea Straniero, Mariapia Genderini, Massimino D'Armiento, Angela Fumarola, Pierpaolo Trimboli, Patrizia Gargiulo

**Affiliations:** 1 Department of Surgery "Francesco Durante", University of Rome "La Sapienza", Rome, Italy; 2 Department of Experimental Medicine and Pathology, Chair of Endocrinology, University of Rome "La Sapienza", Rome, Italy; 3 Department of Clinical Sciences, University of Rome "La Sapienza", Rome, Italy

## Abstract

**Background:**

Thyroid size is a very important criteria of MIVAT exclusion because the working space provided by the technique is limited.

The aim of this work has been to verify the suitability of MIVAT and its applicability in clinical practice, not only in patients with a thyroid volume up to 25 ml but also in patients with a thyroid volume included from 25 to 50 ml.

**Methods:**

From January 2003 to February 2006, 33 patients have been selected for MIVAT. A completely gasless procedure was carried out through a central 20 to 35 mm skin incision performed "high" between the cricoid and jugular notch.

**Results:**

The patients were separated in 2 groups. The first group (less than 25 ml) included 23 patients, the second group (from 25 to 50 ml) included 10 patients. The skin incision performed was from 20 to 25 mm (mean 23.61 mm ± 1.83) long in the first group and from 25 to 35 mm (mean 27.8 mm ± 2.20) long in the second one; this difference is significant (t test p < 0.001).

**Conclusion:**

Our study suggest that the MIVAT using for thyroids bigger than 25 ml and up to 50 ml in volume is feasible and safe. This way allows more patients, excluded before, to take the advantages of minimally invasive approach.

## Background

The targets of minimally invasive video-assisted thyroidectomy (MIVAT) could be summarised by: achievement of the same results as those obtained with traditional surgery, less trauma, better postoperative course, early discharge from hospital and improved cosmetic results.

After the first endoscopic parathyroidectomy, performed and described by Gagner in 1996 [1], several surgeons reported their experiences with minimally invasive and video-assisted surgery of the neck [2-24]. Shimizu successfully treated more than 120 patients using the anterior neck lifting method [2,3]. Ikeda particularly devoted himself to an endoscopic procedure by anterior chest approach [4-7] and Ohgami by breast approach [8]. Miccoli confirmed the suitability of MIVAT by a 15–20 mm transversal skin incision made 2 cm above the sternal notch [9-16].

The patients were considered eligible on the basis of the following criteria: single nodule or small goiter (toxic or not), cranio-caudal axis of the lobes not longer than 7 centimetres, the largest transversal diameter not exceeding 3.5 centimetres, small (max 2 cm) papillary carcinoma without lymph node involvement.

Thyroid size bigger than 25 ml is an important cause of exclusion, because the working space provided by the technique is limited.

**Figure 1:**
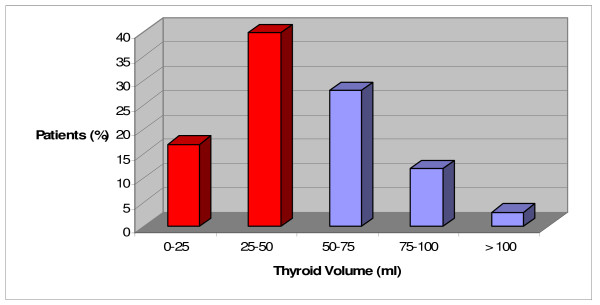
Rationale of the work.

We observed, in our last 100 patients undergone a thyroid exeresis, that thyroid size, echographically measured, is frequently included from 25 to 50 ml and that this group represents about the 40% of total (fig. 1). The MIVA approach now excludes these patients.

The aim of this work has been to verify its suitability and its applicability in clinical practice, not only in patients with a thyroid volume up to 25 ml but also in patients with a thyroid volume included from 25 to 50 ml. In our opinion this approach is possible in these cases by a small increase of skin incision. This way allows more patients, excluded before, to take the advantages of minimally invasive approach.

## Methods

### Patients

In our Department from January 2003 to February 2006 33 patients (31 females and 2 males) have been selected for MIVAT. Preoperative evalutation has been obtained in all cases. Determination of thyroid size has been calculated by sonographic methods. An our study on this procedure, about its details and accuracy, is actually in progress. This study, like other studies, demonstrates that the preoperative measurement of the thyroid gland by ultrasound is underestimated in comparison to the real volume of the excised gland; nevertheless ultrasound is more reliable in symmetrical abnormalities of thyroid gland. In order to obtain a more correct preoperative evaluation, the best way would be to search for a mathematical model that takes into account the irregular shape of an abnormal thyroid gland [26].

In this retrospective study our patients were separated in 2 groups. The first group included patients with a thyroid volume less than 25 ml (17.09 ml ± 4.71) and the second patients with a thyroid volume from 25 to 50 ml. (34.6 ± 6.59)

### Surgical instruments

The requested instruments for this kind of surgery are in part the same used for the traditional one; moreover, this technique utilizes proper tools with small diameter: atraumatic spatulas, spatula-shaped aspirator, forceps and scissors. Nevertheless for MIVAT the primary instruments are the 30-degree 5-mm endoscope and the 14 cm-long Harmonic Scalpel Scissors (Ethicon ENDO-SURGERY, Inc.).

### Surgical procedures

In our experience we adopted a "modified Miccoli-procedure".

**Figure 2:**
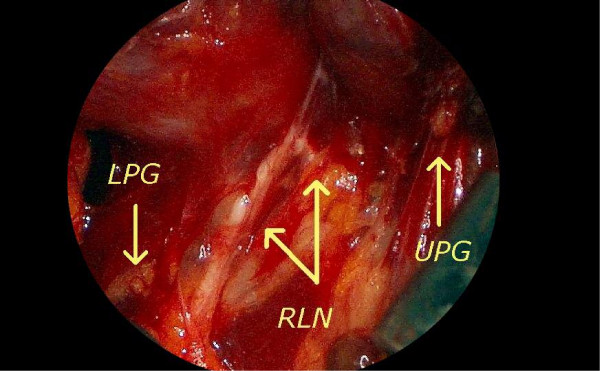
Endoscopic identification of the left recurrent laryngeal nerve (RLN), upper (UPG) and lower (LPG) parathyroid glands.

We can resume the MIVAT in following five steps:

1. High cervical skin incision and access to the operative space

2. Endoscopic dissection of the upper pedicle with identification and preservation of the external branch of the superior laryngeal nerve (EBSLN)

3. Endoscopic identification of the recurrent laryngeal nerve (RLN) and parathyroids glands (fig. 2)

4. Extraction and resection of the lobe

5. Closure

The neck is little hyperextended. The surgical team consists of the surgeon and three assistants, one of whom must hold the camera. A 20–35 mm skin incision is performed "high" between the cricoid and jugular notch, in the middle line on account of its upper migration when the patient is placed in the supine position [25].

**Figure 3:**
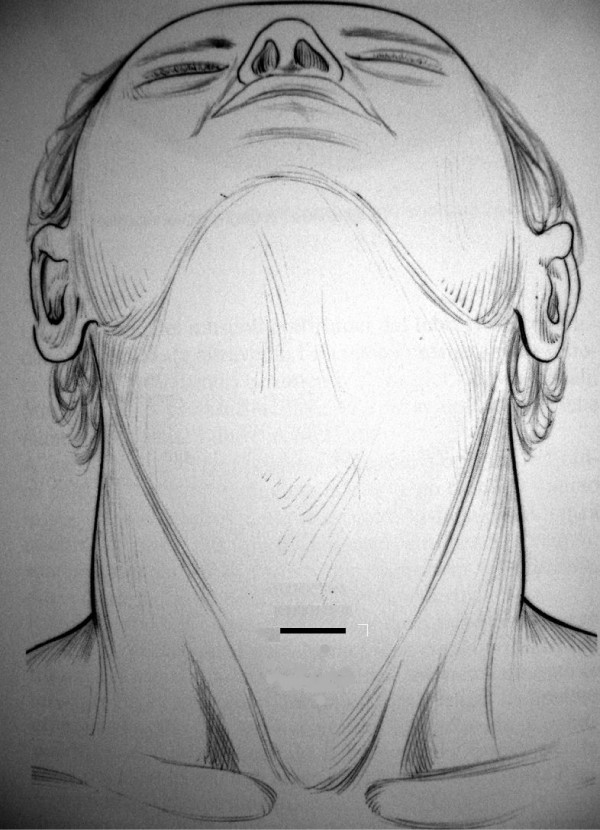
A 20–35 mm skin incision is performed "high" between the cricoid and jugular notch, in the middle line.

In our opinion this kind of "high"scar on the neck results mostly hidden by the curve of the neck and by the shade of the chin (fig. 3).

The cervical linea alba is then opened. The thyroid lobe on the affected side is then carefully dissected from the muscles. Two small retractors are used to medially retract and lift up the thyroid and to laterally retract the muscles to maintain the operative space. A 30-degree 5-mm endoscope is inserted through the skin incision.

The area must be completely bloodless, because even minimal bleeding makes the operation more difficult or impossible. To achieve haemostasis, we use the 5 mm, 14 cm-long Harmonic Scalpel Scissors. The first vessel to be cut is the middle vein, when present, or the small veins between the jugular vein and the thyroid capsule. The spatula is used to separate the larynx from the vessels and to retract them laterally. The EBSLN can be easily identified after dissection of the different components of the upper pedicle. The upper pedicle is then exposed and selectively cut by Harmonic Scalpel Scissors. In some cases it's possible to use previously an adsorbable tie.

The inferior vessels are also clipped and cut off, exposing the antero-lateral side of the trachea. The RLN generally lies in the thyrotracheal groove, behind the Zuckerkandl tubercle. The RLN and the parathyroid glands are visualized by endoscope magnification [17]. Now the operation is conducted as in open surgery: the lobe is freed from the trachea, the isthmus dissected from the trachea and divided by Harmonic Scalpel. Finally the lobe is removed by conventional open technique. For total thyroidectomy, the same technique is repeated in the controlateral side. Spray "fibrin glue" sealants are applied and only one drainage tube (2.33 mm) is laterally introduced.

The muscles incision is sutured with absorbable suture and the wound is closed by intradermic adsorbable suture. All patients, in the postoperative care, take a calcium and vit.D gradual decreasing supplementation to avoid the possible but not important transitory hypoparathyroidism caused by postoperative local edema. Blood tests have been performed in the first postoperative day and, after discharged, at one week from surgery.

In the postoperative stay, all patients were asked to evaluate the pain that feel by means of a numeric scale. This scale ranged from 0 to 10 (where 10 is maximum pain).

With the same method, at 6 months after operation, all the patients were asked to evaluate the cosmetic result of the procedure (where 10 in the scale was most excellent cosmetic result).

## Results

The first group includes 23 patients, the second group includes 10 patients. The skin incision performed was from 20 to 25 mm for the patients belonging to the first group (mean 23.61 mm ± 1.83) and from 25 to 35 mm for the patients to the second group (mean 27.8 mm ± 2.20); this difference is significant (t test p < 0.001).

In the first group we performed 20 total thyroidectomy and 3 hemithyroidectomy. Only in one case conversion to the traditional approach has been necessary for difficulties in finding the RLN. No complications have happened, except for 2 cases of transitory (two months) hoarseness at the beginning of our experience. The operative average time for total thyroidectomy has been 134.35 minutes ± 13.43

We did not observe definitive hypoparathyroidism after discharged at the suspension of supplementations therapy.

In the second group we performed 9 total thyroidectomy and 1 hemithyroidectomy. No conversion to traditional approach has been necessary and no complications have happened. The operative average time for total thyroidectomy has been 110 minutes ± 14.91. The difference among groups is significant (t test p < 0.001).

The slight difference of surgical mean time between the two groups could be explicable because in the second group the enlargement of the skin incision make the endoscopic identification and surgical dissection of the RLN easier.

In the situations of no clinical evidence of dysphonia, postoperative laryngoscopy wasn't performed in all cases because the cost wasn't justified. In our study we have prescribed postoperative laryngoscopy only in the 2 cases of clinical evidence of hoarseness and in one case with complex surgical act. For these reason no objective assessment of the incidence of recurrent nerve damage is available for this study.

All patients were discharged from the hospital at the second day after surgery once the dreinage tube was removed.

We obtained in both groups excellent results about patients cure rate and comfort, with short hospital stay, few postoperative pain and attractive cosmetic results. The postoperative pain was less if compared with the patients underwent a traditional thyroidectomy. Mean score for pain was often negligible (from 0 to 1.0 in the scale) 24 hours after surgery, while with traditional thyroidectomies performed in the same period in our Department the score was from 1.0 to 3.0 after 24 hours.

The cosmetic result was consider excellent by most patients underwent MIVAT. Mean score after 6 months was 8.9 on a scale of 0 to 10.

Finally, histological exams reveals, for the first group, diagnosis of multinodular goiter in 15 cases, toxic adenoma in 2 cases, papillary carcinoma in 5 cases and thyroiditis of Hashimoto in 1 case; for the second group, diagnosis of multinodular goiter in 7 cases, toxic adenoma in 1 case, papillary carcinoma in 2 cases.

## Discussion

Nowadays MIVAT, in selected patients, clearly demonstrates excellent results regarding patient cure rate and comfort, reduces postoperative pain with shorter hospital stay and more attractive cosmetic results expecially for young female patients. If compared to conventional surgery, MIVAT reduces the invasivity of the surgical manoeuvres and avoids the paresthetic consequences of the superior miocutaneal dissection.

Thyroid size is an important cause of MIVAT exclusion, because the working space provided by the technique is limited. So far MIVA approach was performed in patients with thyroid volume less than 25 ml [9-16] or 30 ml [19-21] with skin incision up to 25 mm. The "traditional open neck surgery" was performed in remaining patients.

Since in our last 100 patients undergone a thyroid exeresis we observed that the rate of gland size up to 50 ml was above 50%, we checked the suitability of MIVAT and its applicability in clinical practice as well as in patients with a thyroid size from 25 to 50 ml. This way allows more patients, excluded before, to take the advantages of minimally invasive approach. The total thyroid volume, echographically determined, must not exceed 50 ml.

We did not found considerable differences of results in the groups of this study and no substantial differences of complications rate with the traditional open neck surgery. In our series we have found instead that the postoperative haematoma rate is lower than the one with traditional thyroidectomy. We had some difficulties "time consuming" in third step in both groups.

In literature, in T1 papillary thyroid carcinoma, the completeness obtained with MIVAT is similar to that obtained with open thyroidectomy in terms of completeness of the surgical resection, as demonstrated by postoperative serum thyroglobulin (Tg) measurements, ultrasound scan and total body scintigraphy [15,19]. In addition, this technique offers all the great advantages of a minimal neck wound [15,19]. No conclusions can be drawn in terms of influence of MIVAT on the outcome of the patients with small papillary thyroid carcinoma. However a higher number of cases and a longer follow-up are needed to confirm the safety of this procedure on the management of papillary cancer of the thyroid [27].

## Conclusion

Therefore, the aim of this work has been to verify the suitability of the MIVAT in clinical practice, by the widening of the thyroid size criteria, because this approach is still indicated only for a minority of patients.

Our study suggests that the MIVAT using for thyroids bigger than 25 ml and up to 50 ml in volume is feasible and safe.

We observed that by a small increase of skin incision, it's possible to significantly raise the number of patients eligible for the MIVA-technique and allow more patients to take advantage of minimally invasive approach. We think that, when possible, this procedure should be performed.

## Competing interests

The author(s) declare that they have no competing interests.

## Authors' contributions

All Authors contribuited likewise in this study.

All Authors read and approved the final manuscript.

## Pre-publication history

The pre-publication history for this paper can be accessed here:


